# On the Treatment of Paralysis

**Published:** 1870-07

**Authors:** 


### On the Treatment of Paralysis.

A. D. Rockwell, M. D., of New York, in a paper read recently before the New York Academy of Medicine, "On the Treatment of Paralysis by Electrization," sums up the leading ideas advanced, as follows: 1st. Paralysis is not a disease, *per se*, but is merely a symptom of some disturbance of the central or peripheric nervous system. The treatment of paralysis should have reference not alone to the affected parts, but also to the general condition of which it is the result, or with which it is associated. 2d. Paralysis—throwing aside the toxic, traumatic, and local varieties—is pre-eminently a symptom of exhaustion or weakness of the nervous system. It occurs most frequently among the cultivated and the intellectual. It is a malady of the two extremes of life—infancy and old age. 3d The two cardinal principles of treatment of paralysis are, in the first place, to administer remedies directed to the removal of the cause, that is, the condition of the system, or the local injury of which the paralysis is a symptom; and, in the second place, to restore the tone and nutrition of the affected parts. The first condition is met by the administration of strychnia (internally or hypodermically) when the central nervous system is anæmic; in the use of iodide of potassium, ergot, belladonna, and bromide of potass, when the spine or brain is congested or inflamed; and by the use of general tonics, such as bark, iron, phosphorus, arsenic, and oxide of zinc. The second condition is met by rubbing, shampooing, etc. Electrization general or localized, with the varying use of the galvanic or faradic currents, fulfils these indications better than any other method of treatment. It benefits paralysis by virtue of its tonic effects on the nervous and muscular system. 4th. In the treatment of paralysis by electrization, the distinction between the effects of the galvanic and faradic currents *is of radical and indispensible importance.* An intense, penetrating galvanic current, from 50 or 100 cells of Chester's air-tight battery will overcome far greater resistance than the faradic; will produce muscular contractions in paralyzed limbs when the faradic is powerless, and will also more directly and positively effect the great sympathetic. 5th. Fresh recent attacks of a local character are sometimes very quickly relieved and cured by the faradic current alone; but long-standing and severe cases, that constitute the majority, demand the varying and preserving use of both the galvanic and faradic currents for weeks and months. 6th. The prognosis is far more favorable than is commonly supposed. During the past two years he has treated by electrization, general and local, and by the varying use of the faradic and galvanic currents, seventy cases of paralysis, resulting from a variety of causes, and manifesting itself in different parts of the body. Of these 70 cases, 16 were supposed to result from cerebral effusion, causing hemiplegia; of this number 11 suffered from paralysis of the right, and 6 of the left side; 3 of the cases were approximately and 2 completely cured; 3 were very markedly benefited; 3 discontinued treatment after a few visits; in three cases only slight amelioration was evident, while two 2 failed to receive any benefit whatever.

Four cases of complete facial paralysis, 2 of which involved the right side, 1 of the left, and 1 both sides of the face.

With the exception of the case involving the 7th pair on either side following siphilis, they were all quickly and completely cured. Anæsthesia occured twenty times. In nine cases the cure was complete; nine were approximately cured, while two discontinued treatment before any result could be seen. Four cases of complete paraplegia, three of which received no benefit whatever, while 1 was approximately cured after a protracted course of treatment. One of the patients died while under treatment.

Eight cases of infantile paralysis, probably of a reflex character. Four of these patients were completely and rapidly cured, and two were approximately cured, while two received no benefit whatever.

Twelve cases of paralysis were treated, each of which involved but one limb of the body. One of these was approximately and five completely cured; four derived marked benefit, while two discontinued treatment before any result was noticed.

Four cases of palsy agitans, but in one case only did Dr. R. succeed in obtaining even a slight amelioration of the symptoms. Writer's cramp occured twice. One patient recovered considerable power in the affected hand, while the other experienced no relief worthy of mention —*Medical Record.*

